# A probabilistic bridge safety evaluation against floods

**DOI:** 10.1186/s40064-016-2366-3

**Published:** 2016-06-18

**Authors:** Kuo-Wei Liao, Yasunori Muto, Wei-Lun Chen, Bang-Ho Wu

**Affiliations:** Department of Civil and Construction Engineering, National Taiwan University of Science and Technology, No. 43, Sec. 4, Keelung Rd., Taipei, 106 Taiwan; Department of Civil and Environmental Engineering, Tokushima University, Tokushima, Japan

**Keywords:** Bridge safety, Flood-resistant reliability, MCS, Bayesian LS-SVM

## Abstract

To further capture the influences of uncertain factors on river bridge safety evaluation, a probabilistic approach is adopted. Because this is a systematic and nonlinear problem, MPP-based reliability analyses are not suitable. A sampling approach such as a Monte Carlo simulation (MCS) or importance sampling is often adopted. To enhance the efficiency of the sampling approach, this study utilizes Bayesian least squares support vector machines to construct a response surface followed by an MCS, providing a more precise safety index. Although there are several factors impacting the flood-resistant reliability of a bridge, previous experiences and studies show that the reliability of the bridge itself plays a key role. Thus, the goal of this study is to analyze the system reliability of a selected bridge that includes five limit states. The random variables considered here include the water surface elevation, water velocity, local scour depth, soil property and wind load. Because the first three variables are deeply affected by river hydraulics, a probabilistic HEC-RAS-based simulation is performed to capture the uncertainties in those random variables. The accuracy and variation of our solutions are confirmed by a direct MCS to ensure the applicability of the proposed approach. The results of a numerical example indicate that the proposed approach can efficiently provide an accurate bridge safety evaluation and maintain satisfactory variation.

## Background

In Taiwan, the bridge safety evaluation for floods is often a two-step procedure. The first step is to examine bridge safety through a preliminary inspection evaluation form (PIEF). If the overall assessment score from the PIEF does not meet a predefined standard, the evaluation should proceed to an advanced investigation such as pushover analysis to ensure the safety of the bridge. The PIEF consists of several items that are potential threats for bridge safety. Each evaluated item is allocated a weight to indicate its relative importance. The sum of all of the weights is 100. The items in the PIEF proposed by Chern et al. (2007) include the scouring depth, the foundation type, the attack angle of the river flow, the presence of protective facilities at the river bank and bed and the presence of a dam upstream. Among all of the items, the scouring depth has the highest weight and is considered as the most influential factor. Thus, the goal of this study is to investigate the safety of a scoured bridge. To fulfill this purpose, the strengths of a bridge structure such as the strengths of the pile shear stress, the pile axial stress, the horizontal displacement on the pile head, the soil bearing and the soil pulling force need to be carefully considered. In addition, the corresponding demands for the aforementioned strengths are water surface elevation, water velocity, local scour depth, wind load and soil properties that should be included in the safety evaluation as well. Because the variation of the main channel location is not considered, the worst case scenario is used. In other words, this study does not analyze every pier in a bridge; instead, only the pier that has the highest risk is analyzed. Because a one-dimensional hydraulic model is adopted here, all of the piers in the same river cross section share the same water level and velocity. The pier with the lowest river bed profile is selected for analysis because it has the largest flow depth, resulting in the largest scour depth.

**Fig. 1:**
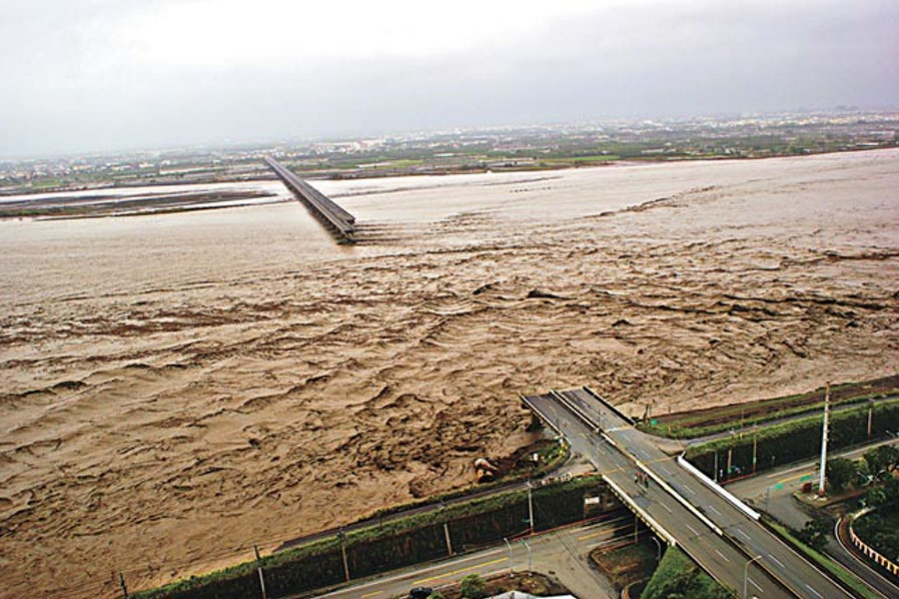
Collapse of Shuangyuan Bridge (2009/8/10) (photo courtesy of Apple Daily)

Currently, bridges in Taiwan are designed against a specific return period (e.g., 100-year flood) that is represented by a set of deterministic numbers (e.g., a fixed stream velocity and level). For this reason, many studies have evaluated bridge safety through a deterministic process. For example, Chern et al. (2007) evaluated the safety of a bridge through a stability examination of the soil bearing capacity, pile strength and bridge serviceability. A deterministic finite element method was used by the National Freeway Bureau in Taiwan (NCREE 2010) and Sung et al. (2011). To further consider the influence of floods, in addition to the bridge model, hydraulics and fluid–solid interaction analyses were included in the safety evaluation (Wang et al. 2011). A recently retrofitted bridge (Shuangyuan Bridge) that failed to survive under Typhoon Morakot (Fig. 1) has drawn the attention of many engineers. Sixteen piers of the Shuangyuan Bridge collapsed. The 24-h accumulated rainfalls of 11 (out of 15) precipitation stations (located in the Gaoping River basin) ranged from 1147 to 1340 mm. Based on a frequency analysis (Water Resources Agency 2009), the return periods are 2000 years and more than 200 years for rainfall and flow discharge, respectively. Apparently, using a deterministic number with a safety factor to design or retrofit a bridge cannot ensure bridge safety against a hazard that has a longer return period than the designated one. Therefore, a probabilistic approach is needed and adopted in the current study.

Many researchers have evaluated bridge safety using a probabilistic approach. For example, Carturan et al. (2012) used a stochastic finite element method to retrieve the reliability of a bridge. Wu et al. (2011) used a most probable point-based (MPP-based) reliability method to evaluate the reliability of a levee system in the Keelung River. Adarsh and Reddy (2013) used advanced first-order and second-moment (AFOSM) and a Monte Carlo simulation (MCS) to evaluate the safety of artificial open channels. Davis-McDaniel et al. (2013) used a fault-tree model to perform bridge failure risk analysis for bridges in South Carolina. The results showed that flood, scour, overloading, corrosion of post-tensioning tendons and earthquakes are the top five critical factors. Saydam et al. (2013) evaluated the bridge superstructure safety via a probabilistic approach, in which the component and system level failures were both considered. Zhao et al. (1997) integrated the linearly constrained MCS with unit hydrograph theory and routing techniques to evaluate the reliability of hydraulic structures.

Although many studies have shown that a reliability analysis is necessary for evaluating bridge safety, few of the studies in Taiwan have adopted this approach. Thus, this study builds a reliability analysis framework to reflect several important bridge safety issues through a case study. For example, only reliability analysis takes the uncertainty or variation of the influencing factors into consideration in the safety evaluation process. Based on the current study, uncertainty plays an essential role in bridge safety. Many issues such as the pile shear stress, the pile axial stress, the pile head horizontal displacement, the soil bearing and the soil pulling force, are involved in a bridge failure; therefore, a system reliability analysis is needed. Thus, the five performance functions above are considered in our bridge system. Among these five performance functions, variables such as the water surface elevation, water velocity, local scour depth, wind load and soil property are pertinent and treated as probabilistic density functions. Although MPP-based approaches are often adopted in a reliability analysis, they are not suitable in the current study because of the nonlinearity and complexity of the analyzed problem. Sampling approaches such as MCS and importance sampling (IS) are potential reliability analysis tools in the current study. However, the cost of such approaches is often unaffordable for a practical problem. Recently, many studies have utilized a response surface in reliability analysis. For example, Sun et al. (2016) analyzed the reliability of a 2.5D/SiC composite turbine blade using a response surface built by support vector machines (SVM). Zhao and Qiu (2013) used the center point of the experimental points to control the construction of their response surface for reliability-based optimization. This study adopts Bayesian least squares support vector machines (LS-SVM, Suykens et al. 2002) to build the response surface, followed by an MCS. Both the accuracy and variation of the proposed approach are ensured by comparing the solution from the MCS.

The five performance functions, the random characteristics of a bridge against floods, the construction of a response surface and the proposed reliability analysis process are described below, followed by a demonstration via a numerical example.

## Descriptions of the performance functions

### Load types

The loads that are considered in this study include the vertical load, wind load and hydrodynamic pressure. The hydrodynamic pressure is calculated according to Eq. (1) (Wu et al. 2014):

1 $$p_{ave} = \frac{{52.5K(V_{ave} )^{2} }}{1000}$$

where *P*_*ave*_ is the average water flow pressure (tf/m^2^), *V*_*ave*_ is the average water velocity (m/s) and *K* is 1.4, 0.7 and 0.5 for flat, round and pointed pier shapes, respectively.

**Fig. 2:**
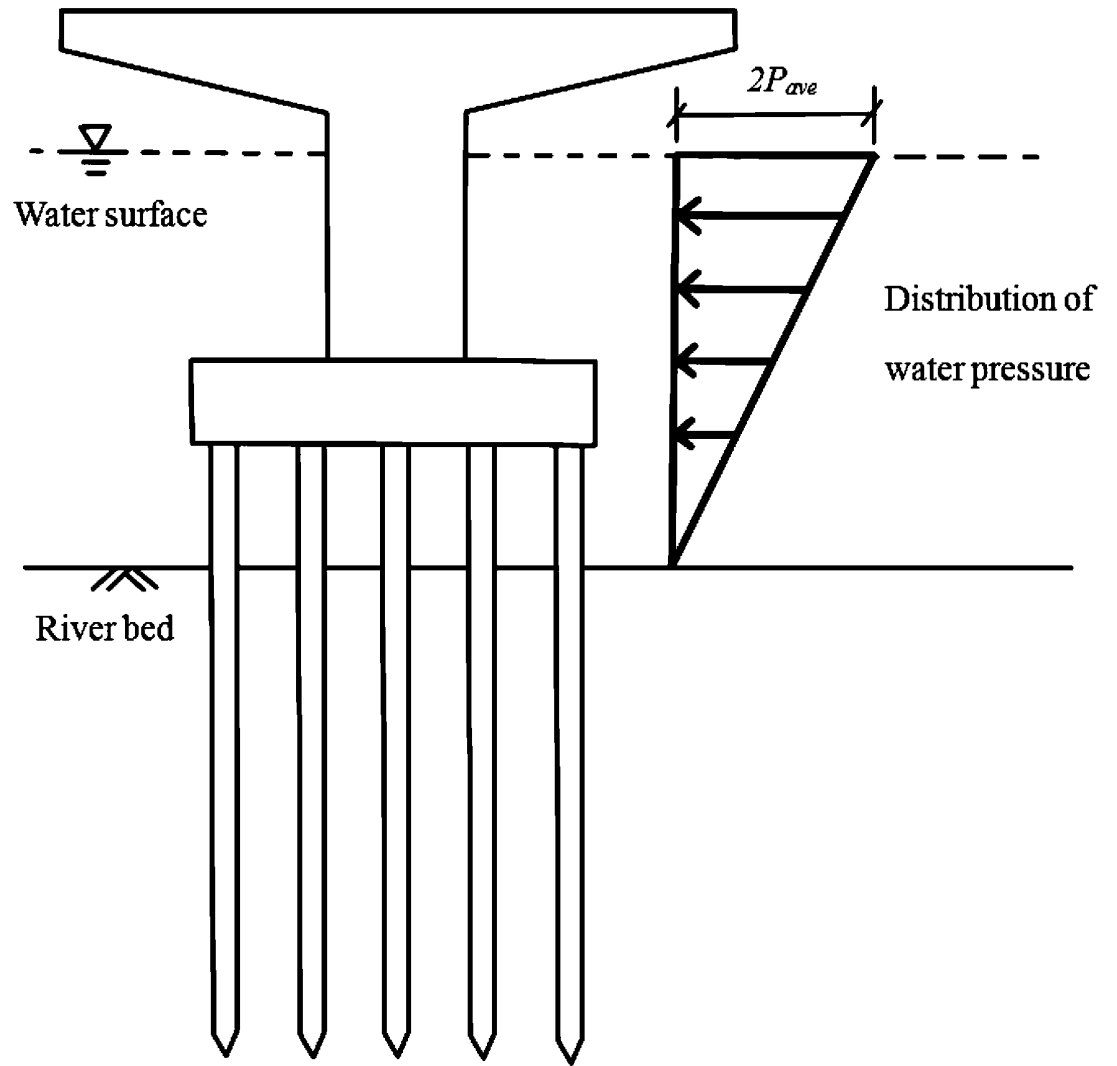
The pressure distribution of water flow

The maximum water flow pressure *P*_*max*_ is twice the average water flow pressure *P*_*ave*_. The water flow pressure is triangularly distributed from the top of the water surface elevation (*P*_*max*_) to the riverbed (zero), as shown in Fig. 2. The hydrodynamic force is the product of multiplying the water pressure by the corresponding projected area on the bridge. If the water flow reaches the bridge PCI girder, then the influence of the hydrodynamic force on the superstructure is also considered.

A deterministic analysis approach (e.g., the analysis procedure provided in the design code) uses the load and reduction factors (i.e., LRFD in steel structure design) to consider the uncertainty in the variables (i.e., loads or material properties). Because this study utilizes reliability analysis to take the uncertainty into consideration, the vertical load (both dead and live loads) and wind load are calculated according to the provisions of “The Bridge Design Specifications” without using the load factors (2009). The bridge that is considered in the numerical example is a 4-lane bridge with a span of 35 ms.

### Performance functions

The five performance functions considered in this study are described in Eqs. (2), (3), (4), (5) and (6) (Liao et al. 2015), which correspond to the performances of the pile shear stress, the pile axial stress, the bridge serviceability (the horizontal displacement on the pile head), the soil bearing and the soil pulling force, respectively.

2 $$f(S_{1} ) = A\tau_{y} - V_{t} e^{ - \lambda x} [\cos (\lambda x) - (1 + 2\lambda h_{0} )\sin (\lambda x)] = 0$$

3 $$f(S_{2} ) = \frac{{I\sigma_{y} }}{y} - \frac{{V_{t} }}{\lambda }e^{ - \lambda x} \left[ {\lambda h_{0} \cos (\lambda x) + (1 + \lambda h_{0} )\sin (\lambda x)} \right]$$

4 $$f(S_{3} ) = 1.5 - 0.01\left( {\frac{{V_{t} }}{{2EI\lambda^{3} }} + \frac{{M_{t} }}{{2EI\lambda^{2} }}} \right)$$

5 $$f(S_{4} ) = A_{s} f_{s} + A_{b} q_{b} - \frac{P}{n \times m} - \sigma A$$

6 $$f(S_{5} ) = w_{p} + \frac{1}{{K_{2} }}A_{s} f_{s} + \frac{P}{n \times m} - \sigma A$$

where *A* is the pile area, τ_*y*_ is the pile shear strength, *V*_*t*_ is the applied shear force on the top of the pile (*tf*), λ = $\sqrt[4]{kD/EI}$ (*m*^−1^), *k* is the horizontal subgrade reaction coefficient (*tf*/*m*^3^), *D* is the pile diameter (*m*), *E* is the elastic modulus (*tf*/*m*^2^), *I* is the pile cross-sectional moment of inertia (*m*^4^), *x* is the distance between the measured point to the top of the river bed, *h*_0_ = *M*_*t*_/*V*_*t*_(*m*), σ_*y*_ is the yielding stress, 1.5 (cm) is the displacement capacity, *M*_*t*_ is the applied bending moment on the pile head, *A*_*s*_ is the pile surface area, *f*_*s*_ is the friction resistance pressure on the surface of the pile, *A*_*b*_ is the area of the pile bottom, *q*_*b*_ is the allowable vertical pressure at the pile bottom, *P* is the applied vertical load, *n* × *m* is the total number of piles, σ is the resulting stress of the outermost pile due to the bending moment, *w*_*p*_ is the pile weight and *K*_*2*_ is 3 for the case of short-term loading and 6 for the case of long-term loading. The on-site standard penetration test N value is used to estimate *f*_*s*_ and *q*_*b*_, as shown in Table 1.

**Table 1:** *f*_*s*_ and *q*_*b*_ estimated by the N values

	Driven pile	Bored pile	Implant-type pile
*f*_*s*_	$\hbox{min} \left[ \hbox{N}/5, 15 \right]$	$\hbox{min} \left[ \hbox{N}/5,15 \right]$	$\hbox{min} \left[ \hbox{N}/5,15 \right]$
*q*_*b*_	30 N	7.5 N	25 N

The demands of the pile strength [Eqs. (2), (3) and (4)] are calculated based on Chang’s simplified lateral pile analysis (Chang and Chou 1989). However, the boundary conditions that are defined in Chang’s method (Chang and Chou 1989) are not exactly the same as in the situation that is considered here. For example, in Chang’s method, the external force is a concentrated force and is applied at the pile head, which is not applicable when scouring occurs, as shown in Fig. 2. To use Chang’s formula, an equivalent force of the hydrodynamic pressure is calculated, for which the detailed description is as follows.

**Fig. 3:**
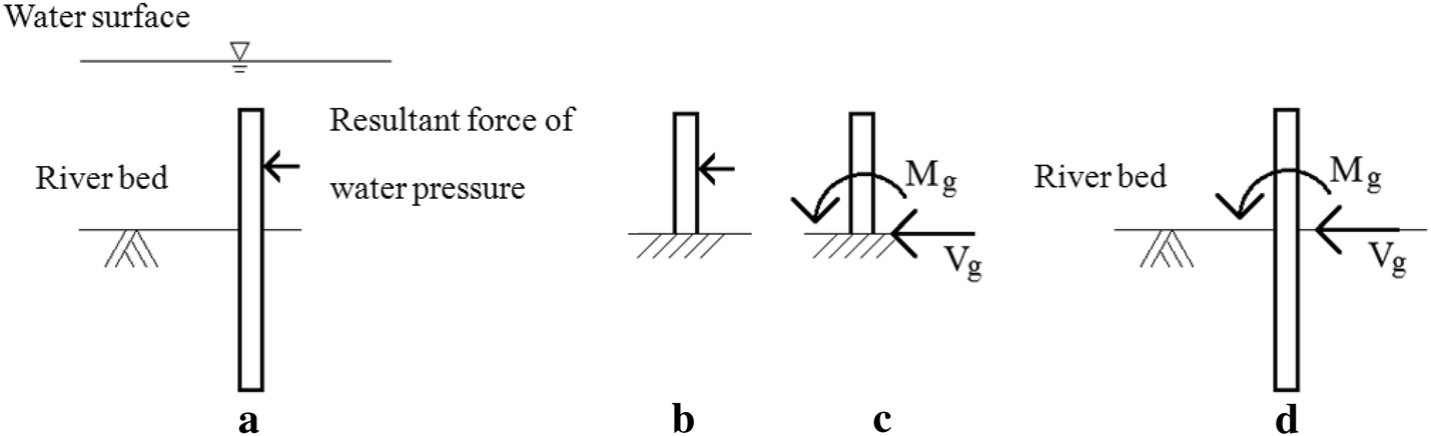
The equivalent force of water pressure when pile head is free: **a** the original pile; **b**, **c** the equivalent pile, **d** pile with equivalent force

**Fig. 4:**
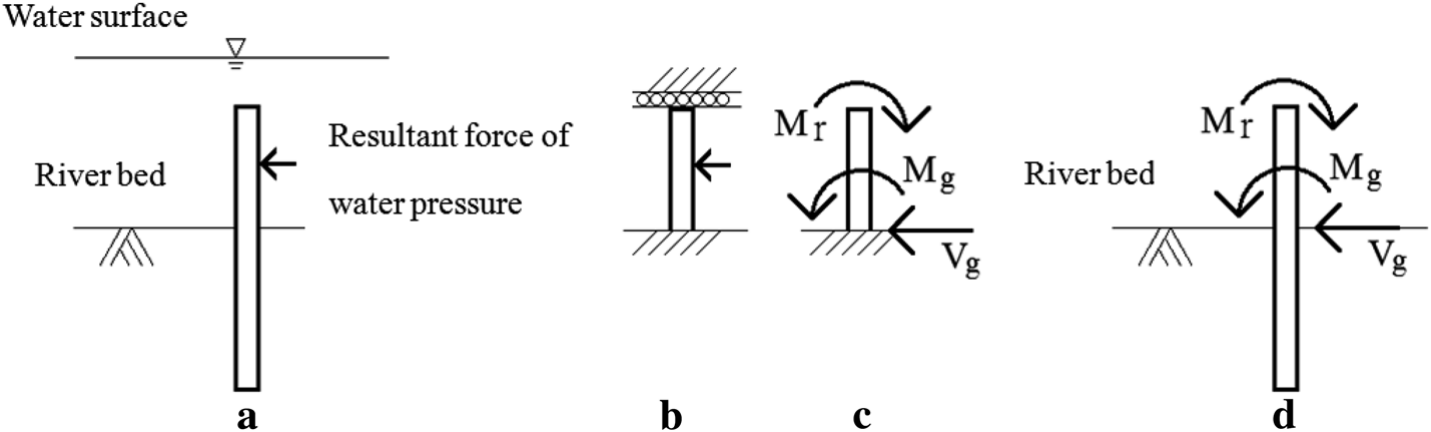
The equivalent force of water pressure when pile head is restrained: **a** the original pile; **b**, **c** the equivalent pile, **d** pile with equivalent force

**Fig. 5:**
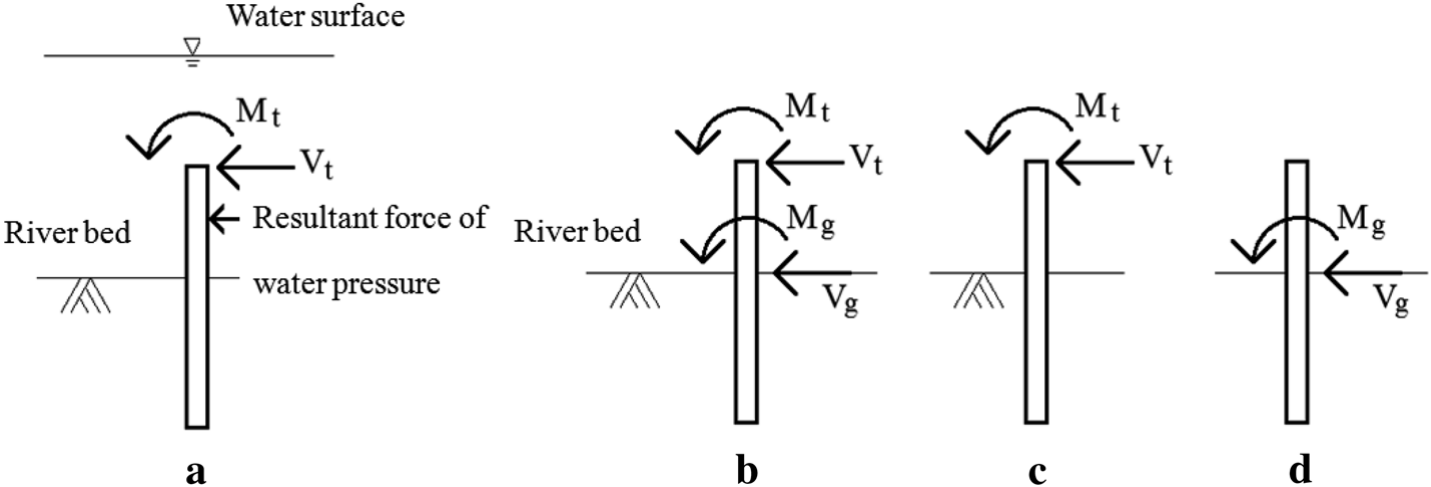
Using superposition to calculate pile demand: **a** the original pile; **b** the equivalent pile, **c** pile with original external force only, **d** pile with equivalent force only

According to Chang’s approach, there are two boundary conditions for the pile head: free or restrained. The boundary condition of the pile head depends on the stiffness of the pile cap. Based on “Building infrastructure design specifications in Taiwan,” if the thickness of the pile cap is less than the pile diameter, then the deformation effect of the pile cap should be considered and is assumed to be free in the current study. On the other hand, if the thickness of the pile cap is greater than the pile diameter, then the pile head is assumed to be restrained. If the pile head is free (Fig. 3), then the equivalent force of the dynamic hydraulic pressure, as shown in Fig. 3d, is estimated by assuming that the boundary conditions at the riverbed surface are fixed, as shown in Fig. 3b, c. A similar approach is applied to the case when the pile head is restrained, as shown in Fig. 4. After obtaining the equivalent force (M_r_, M_g_ and V_g_), superposition theory is used to calculate the demand of the pile axial stress, shear stress and top displacement. Taking the free pile head as an example, to obtain the pile demand, we first convert the original pile (Fig. 5a) to an equivalent pile (Fig. 5b); the pile demand is then calculated by adding the pile demand with the original external force (Fig. 5c) and the pile demand with the equivalent force (Fig. 5d). The pile demand with the original external force is calculated according to the prominent pile equations of Chang’s formula; the pile demand with the equivalent force is calculated according to the embedded pile equations of Chang’s formula ignoring the cantilevered part of the pile. The pile axial stress and shear stress demands are obtained via the superposition theory, as described above. The displacement demand requires further consideration, as illustrated below.

Figure 5d, together with Chang’s formula, provides only the displacement on the riverbed surface. To obtain the pile head displacement, taking the case in which the pile head is free, for example, the pile head displacement is approximately computed according to Eq. (7). If the pile head is restrained, then the pile head displacement is approximately computed according to Eq. (8).

7 $$\delta_{\text{t}} = \delta_{\text{g}} + (h_{\text{t}} - h_{{\text{left}}} )\sin \alpha$$

8 $$\delta_{\text{t}} = \delta_{\text{g}} + \frac{{(h_{\text{t}} - h_{{\text{left}}} )}}{2}\alpha$$

where *δ*_t_: the pile head displacement due to an equivalent force, *δ*_g_: the displacement at the riverbed surface due to an equivalent force, *α*: the angular amount at the riverbed surface due to an equivalent force, *h*_t_: total length of the pile, *h*_left_: pile length embedded in the ground.

## Random characteristics of a bridge failure against floods

The uncertainty of the hydrodynamic pressure is affected by the water elevation and water flow velocity, as described in Eq. (1). Water flow often causes foundation scouring, and the scour depth has a major impact on the bridge safety evaluation. However, calculating or measuring the scour depth is not an easy task. As a result, the scour depth is also a factor that has a high degree of uncertainty. The following uses a simple example to explain the unsuitability of using FORM in bridge reliability analysis. FORM considers the reliability problem to be an optimization problem. The goal is to minimize the distance (*β*) between the limit state function and the origin in U-space, while the random variables must stay on the limit state function. The optimum point is known as the most probable point (MPP). Taking the limit state function of the pile head displacement as an example, its mathematical formula of using FORM is as follows:

9 $$\begin{aligned} & \begin{array}{*{20}l} {Min} \hfill & {\beta = \sqrt {\sum\limits_{{}}^{{}} {X^{2} } } } \hfill \\ {s.t. } \hfill & {g(X) = 1.5 - 0.01\left( {\frac{{V_{t} }}{{2EI\lambda^{3} }} + \frac{{M_{t} }}{{2EI\lambda^{2} }}} \right) = 0} \hfill \\ \end{array} \\ & \quad X_{lb} \le X \le X_{up} \\ \end{aligned}$$

where *β* is the objective function, in other words, the distance between the origin to the limit state function in the U-space; and *X* is the group of design variables, in other words, the random variables in the reliability analysis (e.g., the water flow velocity and scour depth). Here, g(*X*) = 0 is the limit state function, and *X*_lb_ and *X*_up_ are the lower and upper bound of the design variables, respectively.

Using data from the Liugui Bridge as an example, after a simple calculation, the g(*X*) in Eq. (9) can be represented as a function of the random variables, as follows:

10 $$\begin{aligned} g(X) & = 0.015 - \left\{ {\frac{{(0.0061898D^{ - 0.75} h_{\text{scour}} + 1)^{3} + 2}}{{217222.77D^{1.75} }}} \right. \\ & \quad \left[ {F_{\text{w}} + 0.03675\;d_{\text{p}} V_{\text{ave}}^{2} (h_{\text{wt}} - 2)\left( {1 + \frac{{h_{\text{scour}} + 2}}{{h_{\text{wt}} + h_{\text{scour}} }}} \right) + 2.0874\;V_{\text{avg}}^{2} \left( {\frac{{h_{\text{scour}} + 1}}{{h_{\text{wt}} + h_{\text{scour}} }}} \right)} \right] \\ & \quad + \frac{{DV_{\text{ave}}^{2} }}{{1810.19\;(h_{\text{wt}} + h_{\text{scour}} )}}\left. {\left[ {\frac{{0.03675\;h_{scour}^{2} }}{{D^{1.75} }} + \frac{{0.032156\;h_{\text{scour}}^{3} }}{{D^{2.5} }} + \frac{{0.013781\;h_{\text{scour}}^{4} }}{{D^{3.25} }}} \right]} \right\} \\ \end{aligned}$$

where *D* is the pile diameter; *F*_w_ is the wind load (*tf*); *d*_P_ is the pier diameter (*m*); *V*_ave_ is the average water velocity (m/s); *h*_wt_ is the designated water level against a specific return period flood (m); and *h*_scour_ is the scour depth (m). The standard penetration test value (SPT-N value) is not shown in Eq. (10) because a single soil stratum is found from the on-site survey.

On the other hand, the MCS regards reliability analysis as an integral problem, as follows:

11 $$P_{f} = J = \int {_{{D_{f}}}^{ \ldots}} \int f _{\text{X}}(x)dx$$

where *P*_*f*_ is the probability of failure, *D*_*f*_ is the failure domain and *f*_**X**_(*x*) is the probability distribution function of the random variables. The reliabilities of using FORM and MCS are displayed in Table 2. Together with Eq. (10), it is evident that FORM is not an appropriate tool for analyzing bridge reliability. To overcome the shortcomings of using MPP-based methods in a nonlinear problem, many studies have proposed modified versions (Zhao et al. 2007). However, this approach is beyond the goal of the current study. Some detailed random characteristics of the bridge reliability are given as follows:

**Table 2:** Illustration of difference in reliability analyzed by MCS and FORM

Method	*P*_*f*_
MCS	3.64 × 10^−3^
FORM	2.68 × 10^−3^

1. In the calculation of the soil bearing capacity and the pile head displacement demand (δ_D_), *q*_*b*_ (the allowable vertical pressure), introduced in Eq. (5), and the coefficient of the horizontal subgrade reaction (*k*_h_) are required. Here, *q*_*b*_ and *k*_h_ are functions of the value of N, as shown in Table 1 and Eq. (12). In general, the value of N is not a deterministic number from an on-site survey, as illustrated in “SPT-N values and the wind load” section. As a result, the soil bearing capacity and *δ*_D_ should be measured using a probabilistic approach.
  12 $$k_{\text{h}} = \frac{{\text{502}N^{{\text{0}\text{.37}}} + \text{691}N^{{\text{0}\text{.406}}} }}{\text{2}}$$
2. Similarly, *f*_*s*_ in the calculation of the soil pulling capacity is a function of the value of N, as shown in Table 1. Furthermore, because the scour depth is a random variable, the pile length embedded in the ground is also a random variable. As indicated by Eq. (13), the number of soil stratums (*n*) to be considered in the analysis depends on the scour depth. As a result, *F*_*s*_ (the friction resistance force on the pile surface) will become a non-continuous function. Thus, it is inappropriate to use the MPP-based approach to calculate the reliability.
  13 $$F_{s} = A_{s} f_{s} = D\pi \sum\limits_{i = 1}^{n} {} h_{\text{i}} \times \text{min}\left[ {\frac{{N_{\text{i}} }}{\text{5}}\;\text{,}\;\text{15}} \right]$$
  where *h*_i_ is the ith soil stratum thickness (*m*), *N*_i_ is the N value for the ith soil stratum and *n* is the number of soil stratums embedded in the ground.
3. As mentioned earlier, Eqs. (2), (3) and (4) can be applied to only one of Chang’s equations. The applicability of Chang’s equation depends on whether the pile is embedded in the ground or not. In other words, once scouring occurs, Chang’s formula should be changed to meet the required boundary conditions. As a result, the corresponding performance functions should be modified accordingly. In this case, the MPP-based approach is not suitable for reliability analysis.

This study applies response surface methodology (RSM) to replace the existing performance functions to improve the efficiency of an MCS. Before explaining the response surface method that was adopted here, the random variables in this study are introduced, which are the water surface level, water velocity, local scour depth, wind load and SPT-N values.

### Water velocity and water surface level

**Fig. 6:**
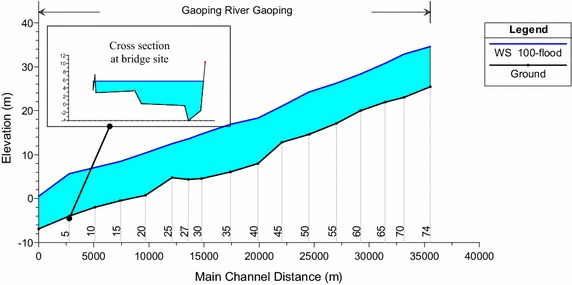
Water surface profile and the analyzed cross section

A probabilistic-based HEC-RAS model is used to investigate the variations and distributions of the water level and water velocity, in which the discharge rate and Manning’s roughness coefficient are considered to be random variables. In HEC-RAS, the water surface levels and velocities are calculated from one cross section to the next by solving the Energy equation. That is, for a given discharge, the water depths and velocities are calculated using an iterative procedure. The distribution types and variations of the discharge rate and Manning’s roughness coefficient are determined as follows. The Manning’s roughness coefficient in the Gaoping River basin is modeled as lnN(−3.395, 0.5508), lnN(−3.571, 0.575) and lnN(−3.802, 0.664) for upstream, midstream and downstream, respectively (Liao et al. 2015). The discharge rate is modeled as N(Q_100_, 0.18^2^) (Sheen 2012). A simulated water level and the cross section of the demonstrated bridge are illustrated in Fig. 6. The distributions of the simulated water level and water velocity are verified by the Chi-square goodness-of-fit test (the p-values are 0.973 and 0.5658 for the water level and water velocity, respectively), indicating that the log-normal distribution is the most suitable distribution for both parameters. From the simulation of HEC-RAS, the coefficient of variations (COV) of the water level and water velocity are 0.135 and 0.35, respectively. The correlation coefficient between the water level and velocity is 0.92. The mean values of the water velocity and water surface are determined according to a 100-year flood suggested by the Ministry of Transportation and Communications R. O. C (2009).

### Local scour depth

**Fig. 7:**
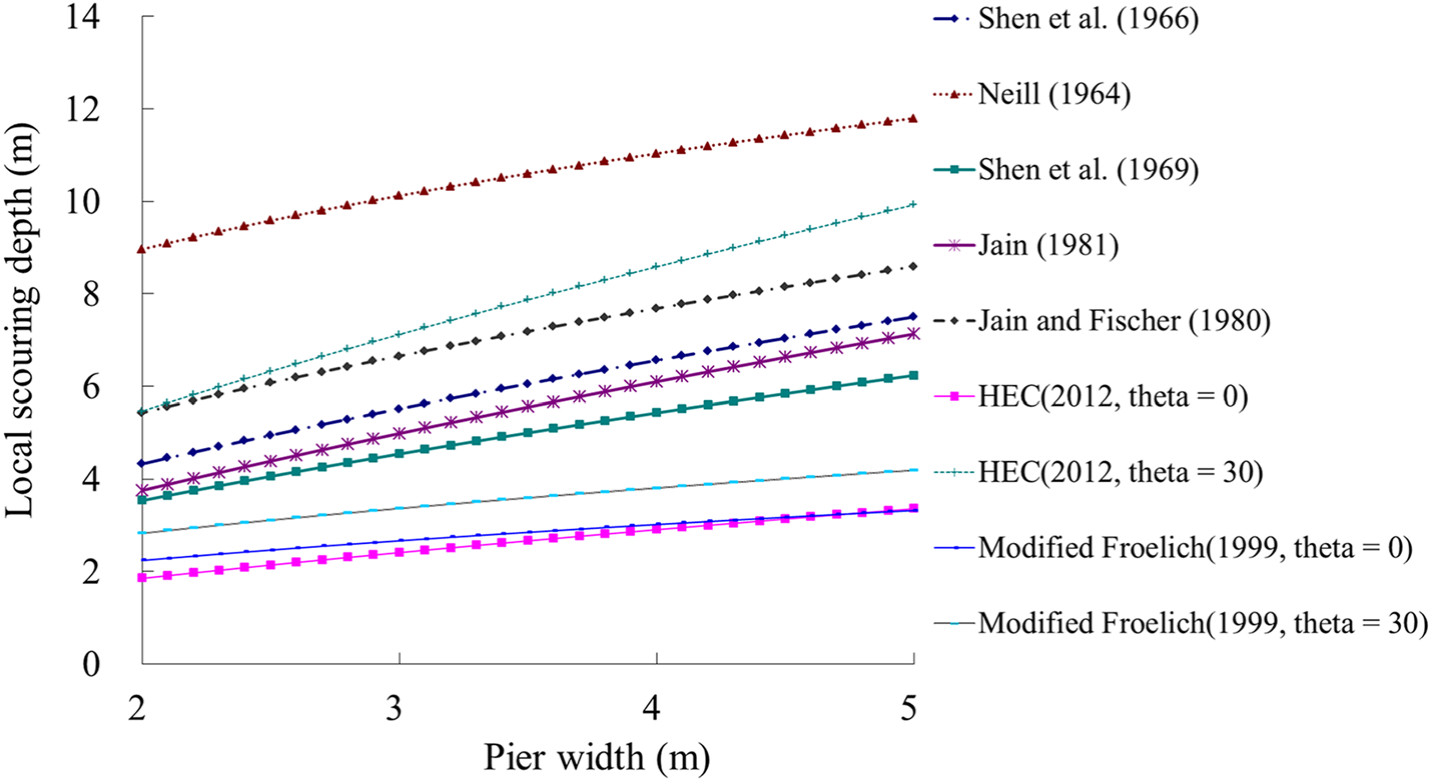
Results of local scour depth using empirical formulae

An empirical formula is often used to determine the local scour depth. Among many well established equations, Li et al. (2011) selected seven formulae for use in Taiwan. Of these seven formulae, five are selected to compute the local scour depth (Neill 1965; Shen et al. 1966, 1969; Jain and Ficher 1980; Jain 1981). The other two formulae suggested by Li et al. (2011) are not included in this study due to a lack of data required for these two equations. On the other hand, two more up-to-date formulae (Fischenich and Landers 1999; HEC-18 2012) are adopted here to improve the prediction accuracy. Thus, this research uses seven empirical formulae to calculate the local scour depth. The scour depth suggested by Fischenich and Landers (1999) and HEC-18 (2012) is a function of attack angle. If the extreme cases are used to determine the attack angle, the resulting values are 0°^0^ and 90°. Here, the extreme cases mean that the selected attack angle will cause the maximum and minimum scour values. However, based on field observation, an attack angle of 90° is rare. In addition, the scour depth with an angle of 30° is approximately 85 % of the scour depth with an angle of 90° for both formulae. Thus, this study uses two attack angles (0° and 30°) to calculate the scour depth for the equations suggested by Fischenich and Landers (1999) and HEC-18 (2012). Because two attack angles for the flow (0° and 30°) are considered in the latter two formulas, nine equations are calculated, as shown in Fig. 7. The figure shows the local scour depth for a bridge that has a pile diameter of 2–5 m. It is observed that Neill (1965) provides the maximum scour depth, while HEC-18 (2012) gives the minimum local scour depth (theta = 0°). Similarly, probability plots and the Chi-square goodness-of-fit test are used to determine the distribution type of the scour depth. The results indicate that the log-normal distribution is suitable for describing the local scour depth for the considered bridge.

Using the 9 scouring depth formulas and 30 pairs of water levels and water velocities, 270 samples of the scour depth are obtained. The sample mean and standard deviation are used as the mean value and standard deviation of the scour depth in the following reliability analysis. The total scour depth of the pier is usually the sum of the depths of the local scouring, contraction scouring and general scouring (i.e., scouring without structures). The local scouring often dominates the overall scouring depth, and thus, other scouring is not considered at present (Alipour et al. 2013; HEC-18 2012; NRCS of USDA 2010). The correlation coefficients between the scour depth and water level and between the scour depth and water velocity are found to be 0.93 and 0.92, respectively.

### SPT-N values and the wind load

The distribution of the SPT-N is summarized according to the geological reports by the Ministry of Transportation, as shown below:

1. The first stratum (immediately below the riverbed): the thickness is approximately 1.3 m, and the SPT-N value has an approximate range of 0–10.
2. The second stratum (below the first stratum): the thickness is approximately 20 m, and the SPT-N value has an approximate range of 2–28.
3. The third stratum (below the second stratum): the thickness is approximately 19.6 m, and the SPT-N value has an approximate range of 3–16.
4. The SPT-N value of the soil stratums below the third stratum is approximately 50.

Because there is no more detailed information for the SPT-N values in the investigated bridge site, the SPT-N values in the first three stratums are assumed to follow a uniform distribution that has the upper and lower bounds indicated above.

Regarding the wind load, the force on the bridge is calculated by multiplying the wind-induced pressure by its corresponding area that is affected by the water level. Once the water level is determined as described in “Water velocity and water surface level” section, the projected area of the wind load can be obtained. The wind load is a random variable because of the uncertainty in the water level.

## The proposed reliability approach and a numerical example

**Fig. 8:**
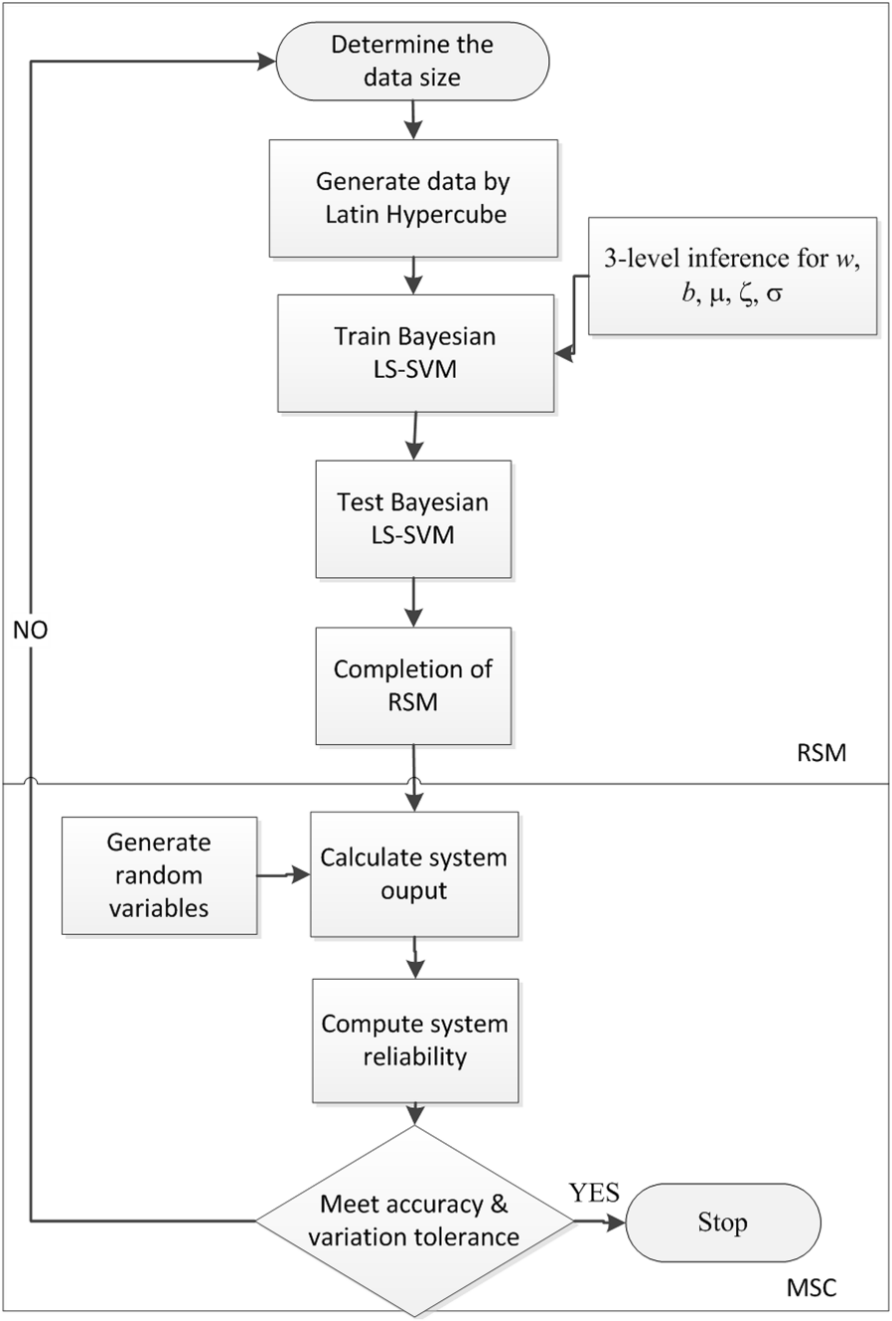
The flowchart of the proposed reliability analysis

A response surface is utilized to calculate the bridge reliability. The proposed flowchart is displayed in Fig. 8. The first step is to construct the response surface, and the second step is to perform the required reliability analysis using MCS. Five parameters of the RSM, which are *w*, *b*, μ, ζ and σ, are determined through a 3-level Bayesian inference, as described below. To confirm the accuracy and efficiency of the proposed algorithm, the results of a direct MCS are used as a baseline. Similar to the classical MCS, the reliability calculated from the proposed approach is a random variable. To ensure the accuracy, this study proposes that the maximum error percentage in a 95 % confidence interval should not be greater than 10 %. Because the MCS solution is a binomial distribution, the aforementioned accuracy requirement will result in a COV of 5 %. Thus, for both MCS and the proposed approach, the COV is limited to 5 %.

### Response surface methodology (RSM)

As shown in Fig. 8, this study first utilizes Bayesian LS-SVM to build the RSM, followed by the classical MCS. The equations needed for constructing a general RSM are described in this section. The purpose of RSM is to replace the performance functions. To fulfill this goal, the equations mentioned in “Descriptions of the performance functions” section are needed in the establishment of the particular RSM in the current study. To be specific, five Bayesian LS-SVM RSMs were built to replace the five performance functions considered here.

A standard SVM, as described in Eq. (14), solves a nonlinear classification problem by means of convex quadratic programs (QP).

14 $$\begin{aligned}\begin{array}{ll} \mathop {\text{minimize}}\limits_{w,b,\xi } & \frac{1}{2}w^{T} w + c\sum\limits_{k = 1}^{N} {\xi_{k} } \\ {\text{Subject}}\,{\text{to}} & \left\{ {\begin{array}{*{20}l} {y_{k} (w^{T} K(x_{i} ) + b) \ge 1 - \xi_{k} } \\ {\xi_{k} \ge 0, \, i = 1,2, \ldots ,N} \\ \end{array} } \right.\\ \end{array} \end{aligned}$$

where *w* is a normal vector to the hyper-plane, *c* is a real positive constant and *ξ*_*k*_ is the slack variable. If *ξ*_*k*_ > 1, the *k*th inequality becomes violated compared to the inequality from the linearly separable case. *y*_*k*_ is the class (e.g., the failure or safe class), [*w*^*T*^*K*(*x*_*i*_) + *b*] is the classifier, *N* is the number of data points and *K* is the kernel function. In the current study, the Gaussian radial basis function (RBF) kernel is used, as shown in Eq. (15).

15 $$K(X,X_{i} ) = e^{{ - \sigma (||X - X_{i} ||)^{2} }}$$

where *X* is the input vector, σ is the kernel function parameter and *X*_*i*_ are the support vectors.

LS-SVM (Suykens et al. 2002), instead of solving the QP problem, solves a set of linear equations by modifying the standard SVM, as described in Eq. (16).

16 $$\begin{array}{*{20}l} {\hbox{min} } \hfill & {\frac{1}{2}w^{T} w + \frac{\gamma }{2}\sum\limits_{k = 1}^{N} {e_{k}^{2} } } \hfill \\ {{\text{s}} . {\text{t}} .} \hfill & {y_{k} (w \cdot K(x_{k} ) + b) = 1 - e_{k} , \quad k{ = 1,} \ldots , {\text{n}}} \hfill \\ \end{array}$$

where γ is a constant number and *e* is the error variable. Compared to the standard SVM, there are two modifications leading to solving a set of linear equations. First, instead of inequality constraints, the LS-SVM uses equality constraints. Second, the error variable is a squared loss function.

Variables such as *w*, *b* and σ in the LS-SVM are determined through Bayesian inference in this study. Bayesian considers undetermined variables as a distribution instead of a single value providing a probabilistic-based classification. The formulation of the LS-SVM is slightly modified, as shown in Eq. (17), for Bayesian inference.

17 $$\begin{array}{*{20}l} {\hbox{min} } \hfill & {\mu \frac{1}{2}w^{T} w + \frac{\varsigma }{2}\sum\limits_{k = 1}^{N} {e_{c,k}^{2} } } \hfill \\ {{\text{s}} . {\text{t}} .} \hfill & { \, y_{k} (w \cdot K(x_{k} ) + b) = 1 - e_{c,k} , \quad k = 1, \ldots ,n} \hfill \\ \end{array}$$

where *c* is the classification and the single regularization constant (γ) in Eq. (16) is replaced by two constants, μ and ζ. To build a response surface using Eq. (17), five unknown parameters (*w*, *b,* μ, ζ and σ) need to be determined. Because the radial base function (RBF) is adopted as the kernel function, σ stands for the width of the kernel. Because of the sequence of determining these unknown parameters, the Bayesian inference adopted here is a 3-level calculation, as shown in Eqs. (18), (19) and (20).

18 $$p(w,b|D,\mu ,\varsigma ,H_{\sigma } ) = \frac{{p(D|w,b,\mu ,\varsigma ,H_{\sigma } )}}{{p(D|\mu ,\varsigma ,H_{\sigma } )}}p(w,b|\mu ,\varsigma ,H_{\sigma } )$$

19 $$p(\mu ,\varsigma |D,H_{\sigma } ) = \frac{{p(D|\mu ,\varsigma ,H_{\sigma } )}}{{p(D|H_{\sigma } )}}p(\mu ,\varsigma |H_{\sigma } )$$

20 $$p(H_{\sigma } |D) = \frac{{p(D|H_{\sigma } )}}{p(D)}p(H_{\sigma } )$$

where *D* stands for the given training data set and *H*_σ_ indicates one attempt of several values of σ for the RBF kernel. It is observed that the primal weight space parameters (*w* and *b*) are inferred at Level 1; the hyperparameters (μ, ζ) are inferred at Level 2; and the RBF kernel parameter is inferred at Level 3.

### Case study

The Shuangyuan Bridge was selected as our case study. Several piers of the Shuangyuan Bridge were seriously damaged during Typhoon Morakot. Thus, the motivation of selecting this bridge is to determine its reliability to provide a more comprehensive explanation of its failure. Information about the bridge before restoration can be found in Liao et al. (2014).

#### MCS reliability and its COV

The MCS solution is a binomial distribution in which its COV with respect to the failure probability is estimated by Eq. (21).

21 $${\text{COV}} = \sqrt {\frac{{1 - p_{f} }}{{np_{f} }}}$$

where *p*_*f*_ is the failure probability and *n* is the sample size. Several MCSs with different sample sizes were conducted to find the necessary number of samples having a COV of 5 %. Table 3 illustrates the relationship between the sample size and COV. It is found that the COV decreased when the number of samples increased, and the sample number corresponding to the target COV was 3000. The system failure probability against a 100-year flood was 2.32 × 10^−1^, which is approximately equal to an annual failure probability of 2.32 × 10^−3^ if the flood is the dominant parameter.

**Table 3:** 100 year failure probability of the selected bridge by MCS

Sample size	100	300	3000
*P*_*f*_	2.34 × 10^−1^	2.30 × 10^−1^	2.32 × 10^−1^
COV	0.181	0.106	0.033

#### Reliability analyzed by Bayesian LS-SVM

As indicted, five performance functions were considered; therefore, five Bayesian LS-SVM RSMs were built. A series system was assumed, and any component failure was considered as a failure of the bridge system. The inputs for each RSM were the five considered random variables and the output was the status of safety or failure. Detailed information for the built RSMs is displayed in Table 4. Please note that this study proposed using Bayesian LS-SVM for RSM construction, indicating that instead of providing a deterministic classification outcome, a posterior class probability was delivered.

**Table 4:** The inputs and outputs of the response surfaces

Inputs	Outputs
Water level	Safety or failure for pile pulling force
Water velocity	Safety or failure for soil bearing force
Local scour depth	Safety or failure for pile shear stress
Wind load	Safety or failure for pile axial stress
SPT-N value	Safety or failure for pile head displacement

Although the RSM has been utilized in many fields, the locations of the sample points significantly influence its accuracy (Zhao and Qiu 2013). Many methods, such as the factorial design, central composite design (CCD), and Latin hypercube design (LHD), are available for engineers to use. Because this study used a computer simulation to analyze bridge reliability, such a process was considered as a computer experiment. The minimum bias design (MBD) was often considered as a good principle for selecting the sample location in this case. The LHD was considered as a space-filling design, focusing inside the design region rather than in the perimeter or the extremes of the design region, and satisfies the criteria of the MBD (Myers and Montgomery 2002). Thus, the LHD was adopted here to construct the RSM for the reliability analysis.

One of the primary goals of this study is to find the optimal sample size of the RSM that has an equivalent solution to that of the MCS. Five sample sizes of 50, 80, 100, 120 and 150 were selected for evaluation. In addition to the sample size, the sample range may influence the reliability calculation. For example, the MCS reliability was 2.32 × 10^−1^, indicating that very few failure points were located outside the ±3σ bounds. Thus, except for the sample size of 150, a sample range of ±2σ was adopted. Although one can compare the RSM reliability with the MCS reliability to examine the accuracy, the root mean squared error (RMSE) was used as an alternative accuracy measurement for the established RSM. Table 5 shows the RMSEs of the five response surfaces with a sample size of 50. It is observed that the highest RMSE was 4.80 %, indicating the built response surface had a good agreement with the original model. Table 6 describes the estimated reliability for the response surface with different sample sizes. As expected, increasing the number of samples reduces the RMSE, resulting in a reliability estimation that is closer to the MCS reliability. Assuming that the allowable error percentage of the reliability estimation is 5 %, among several sample sizes, a sample size of 80 was selected as the optimal size because it has the minimum sample size that satisfied the predefined requirement. Table 7 compares the reliability estimation by the MCS and RSMs with sample sizes of 80 and 150. It was observed that using the RSM can effectively reduce the computational cost for evaluating the bridge reliability.

**Table 5:** The RMSEs of the 5 response surfaces with a sample size of 50

No. of response surface	RSM	RMSE (%)
1	Pile pulling force	4.80
2	Soil bearing force	4.72
3	Pile shear stress	3.30
4	Pile axial stress	3.48
5	Pile head displacement	3.45

**Table 6:** System 100 year failure probability (*P*_*f*_) and accuracy measurements (RMSE) using the response surface with different sample sizes

Sample size	Sample range	*P*_*f*_ (LS-SVM)	*P*_*f*_ (Bayesian LS-SVM)	RMSE (%)
				#5
50	*μ* ± 2*σ*	2.57 × 10^−1^	2.51 × 10^−1^	3.45
80	*μ* ± 2*σ*	2.42 × 10^−1^	2.41 × 10^−1^	1.10
100	*μ* ± 2*σ*	2.38 × 10^−1^	2.37 × 10^−1^	0.65
120	*μ* ± 2*σ*	2.34 × 10^−1^	2.34 × 10^−1^	0.45
150	*μ* ± 3*σ*	2.33 × 10^−1^	2.32 × 10^−1^	0.32

**Table 7:** Comparison of 100 year failure probability (*P*_*f*_) using response surface and MCS

Approach	Bounds^a^	*P*_*f*_	COV
MCS	NA	2.32 × 10^−1^	0.033
RSM(150)^b^	*μ* ± 3*σ*	2.32 × 10^−1^	0.01
RSM(80)^b^	*μ* ± 2*σ*	2.41 × 10^−1^	0.02

^a^Lower and upper bounds for samples to generate the RSM

^b^Sample size

**Fig. 9:**
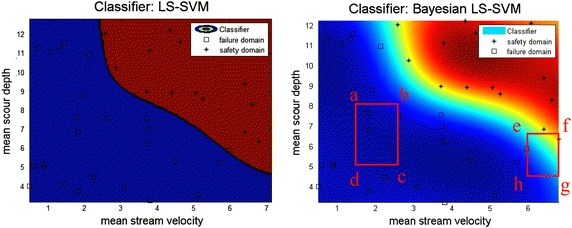
Two established classifiers for the pile head displacement

**Fig. 10:**
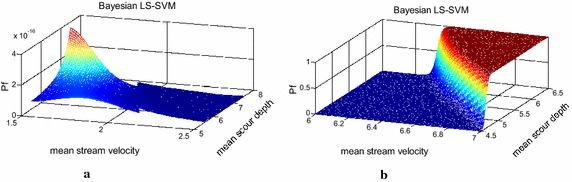
Detailed information for the Bayesian LS-SVM classifier in Fig. 9. **a** Square abcd, **b** square efhg

Table 6 also shows the reliabilities estimated by the LS-SVM. It is observed that using the LS-SVM or Bayesian LS-SVM had only a small difference in the reliability calculation. However, the variation of the reliability solution was significantly reduced by using the Bayesian LS-SVM. Taking a sample size of 50 as an example, the COVs were 0.09 and 0.03 for the LS-SVM and Bayesian LS-SVM, respectively. In this particular case, using a moderate classification successfully reduced the prediction variation. Figure 9 shows two classifiers (LS-SVM and Bayesian LS-SVM) for the pile head displacement performance function. Please note that the established response function consisted of five variables. Only the water velocity and local scour depth are included in Fig. 9. Nevertheless, it is clear that this serviceability performance was highly nonlinear. Figure 9 shows the difference between the deterministic and probabilistic classifiers. The Bayesian LS-SVM, called a moderate prediction machine, delivered an outcome with a probability between 0 and 1, as shown in Fig. 9 (right). The detailed results of the Bayesian LS-SVM (square abcd and square efgh in Fig. 9) are displayed in Fig. 10.

Please note that the COVs of the MCS (the values in Tables 3 and 7) were computed according to Eq. (21). The COVs of the proposed approaches, however, did not follow the binominal distribution. Thus, the COVs of the proposed approaches (the values in Table 7) were calculated from 50 simulations (i.e., performing the MCS 50 times using the established RSM).

Based on the aforementioned computation and discussion, using a response surface with a sample size of 150 can deliver accurate and efficient bridge reliability against floods. To be specific, the sample size was reduced from 3000 to 150. The computational cost can be further reduced to a sample size of 80 if a 5 % error tolerance is allowed in both the accuracy and variation.

Based on the MCS solution, the failure probability of the selected bridge was 2.3 × 10^−1^, which was greater than the threshold value (1.00 × 10^−3^) suggested by the International Organization for Standardization (ISO), indicating that this bridge did not have sufficient reliability, which was consistent with the failure event observed in the Marokot floods. When the proposed algorithm was designed, the sample size and range were two factors that could impact the reliability estimation. Several trial and error tests were conducted to find the appropriate sample size. To determine the sample range, it is intuitive to select a range that basically covered all possible values of the considered random variables. For example, using μ ± 3σ can cover 99.73 % of the possible values of the corresponding variables. However, because the 100-year failure probability of the considered bridge was not a small number, a sample range of μ ± 3σ could be too broad for the current probability level. Thus, a narrower sample range (e.g., μ ± 2σ) was adopted to perform the RSM-based reliability calculation, as shown in Tables 6 and 7. If a 5 % error tolerance was applicable for the accuracy and variance, this approach could effectively reduce the required sample size from 150 to 80.

## Conclusions

A deterministic bridge design or evaluation process is often adopted in Taiwan. After the Morakot Typhoon, engineers realized that a probabilistic approach is needed to consider the uncertainty in the parameters. Therefore, this study builds an accurate and efficient reliability methodology to fulfill such a need. Bridge failure is a complicated system problem, and many different types of events should be considered. Based on the literature and PIEF suggested by an earlier study, the safety of a bridge substructure is one of the most pertinent factors in bridge reliability and is the scope of this study. The random variables considered include the water surface elevation, water velocity, local scour depth, wind load and soil property. A probabilistic hydraulic analysis and on-site survey data are used to capture the variation in these variables. The Bayesian LS-SVM is adopted to establish the response surface, in which the LHS is used to generate the samples. Compared with results from a direct MCS, the accuracy and variation of the proposed method is confirmed. In addition, the reliability obtained from the proposed algorithm for the case study indicated that the selected bridge does not have sufficient reliability, which is consistent with the failure event observed in the Marokot floods. From the reliability derivation for a simplified case and the classifier outcomes, it is observed that the limit state functions are likely to be highly nonlinear and that the sampling method is a suitable choice for reliability analysis. The MCS analysis, however, is time consuming. In the presented case, 3000 samples are needed. The proposed response surface-based reliability analysis can improve the computation efficiency with the same accuracy and variation of the traditional approach (MCS). For example, the sample size is reduced from 3000 to 150. If a 5 % error tolerance is applicable, the proposed approach can further reduce the sample size to 80.
